# HEY2, a target of miR-137, indicates poor outcomes and promotes cell proliferation and migration in hepatocellular carcinoma

**DOI:** 10.18632/oncotarget.9343

**Published:** 2016-05-13

**Authors:** Dan-Chun Wu, Mei-Fang Zhang, Shu-Guang Su, Heng-Ying Fang, Xue-Hua Wang, Dan He, Yuan-Yuan Xie, Xu-Hui Liu

**Affiliations:** ^1^ Department of Rheumatology, The Third Affiliated Hospital of Sun Yat-sen University, Guangzhou, China; ^2^ Department of Pathology, Sun Yat-sen University Cancer Center, Guangzhou, China; ^3^ Department of Pathology, The Affiliated Hexian Memorial Hospital of Southern Medical University, Guangzhou, China; ^4^ Department of Nursing, The Third Affiliated Hospital of Sun Yat-sen University, Guangzhou, China; ^5^ Department of Hepatobiliary Surgery, The Third Affiliated Hospital of Sun Yat-sen University, Guangzhou, China; ^6^ Department of Pathology, The Third Affiliated Hospital of Sun Yat-sen University, Guangzhou, China; ^7^ Department of Emergency, The Third Affiliated Hospital of Sun Yat-sen University, Guangzhou, China

**Keywords:** HEY2, miR-137, prognosis, cell growth, hepatocellular carcinoma

## Abstract

HEY2, a bHLH transcription factor, has been implicated in the progression of human cancers. Here, we showed that HEY2 expression was markedly increased in HCC, compared with the adjacent nontumorous tissues. High HEY2 expression was closely correlated with tumor multiplicity, tumor differentiation and TNM stage. Kaplan-Meier analyses revealed that HEY2 expression was significantly associated with poor overall and disease-free survival in a training cohort of 361 patients with HCC. The prognostic implication of HEY2 was validated in another cohort of 169 HCC patients. Multivariate Cox regression model indicated HEY2 as an independent factor for overall survival in HCC (Hazard ratio = 1.645, 95% confident interval: 1.309-2.067, *P*<0.001). We also demonstrated that HEY2 expression was inhibited by miR-137. In clinical samples, HEY2 expression was reversely associated to miR-137 expression. Furthermore, overexpression of HEY2 increased cell viabilities, colony formation and cell migration, whereas knockdown of HEY2 resulted in the opposite phenotypes. Collectively, our data suggest HEY2 as a promising biomarker for unfavorable outcomes and a novel therapeutic target for the clinical management of HCC.

## INTRODUCTION

The incidence and mortality of hepatocellular carcinoma (HCC) has not been improved in the last decade worldwide [1]. Numbers of new diagnostic and dead HCC cases were 355,595 and 322,416, respectively, in China in 2011 [2]. Due to the intrahepatic metastases and high-risk recurrence, mortality of HCC is the second most common cause of cancer-related death in men and the sixth in women [3]. Therefore, accumulating interests are focus on developing advanced strategies for HCC diagnosis and clinical treatment. Discovery of novel biomarkers has become one of the promising approaches to improve the outcomes of patients with HCC.

Hairy and enhancer of split-related with YRPW motif protein 2 (*HEY2*, also known as *HESR2*, *HRT2* and *CHF1*), belongs to of the HEY family of basic helix-loop-helix (bHLH) transcription factor [4]. HEY2 exerts biological functions on mammalian organ development. Hey2 deficiency resulted in developmental anomalies, such as ventricular wall thinning and postnatal cardiomyopathic changes [5, 6]. HEY2 was also involved in the postnatal retinal vascular remodeling [7], the differentiation of hair cell [8] and coronary vascular maturation [9]. Although the role of HEY2 in human cancer has not been identified, overexpression of HEY2 has been reported in prostate cancer [10], pancreatic ductal adenocarcinomas [11] and hemangioma [12]. Wittmann et al. showed that HEY2 expression was deregulated in high-risk tumors and associated with tumor relapse in Wilms tumor [13]. Furthermore, high expression of HEY2 in prostate cancer was associated with poor survival and served as an independent prognostic factor [10]. However, the role of HEY2 and its clinical significance in HCC remain elusive.

In this study, the expression of HEY2 in HCC was determined at both mRNA and protein levels in two separated cohorts. The correlation of HEY2 expression and clinical features of patients was examined. The prognostic value of HEY2 in HCC was further evaluated. The upstream regulation of HEY2 and its function on cell proliferation and migration were also investigated.

## RESULTS

### HEY2 expression is increased in HCC tissues

**Figure 1:**
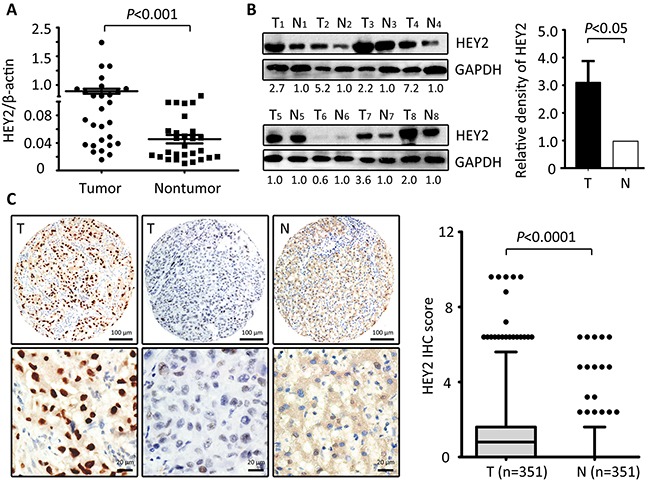
HEY2 expression is increased in HCC **A.** The expression of HEY2 in 28 paired HCC tissues and the corresponding adjacent liver tissues was determined by qRT-PCR (Wilcoxon matched-paired test). **B.** HEY2 protein expression levels were detected by Western blot in 18 HCC samples. The representative images with the ratio of Tumor/nontumor were shown. The relative densities of HEY2 protein in 18 samples was calculated and indicated by histogram. **C.** Representative strong or weak HEY2 immunoreactivities in HCC tissue (T) and negative staining of HEY2 in the nontumorous tissue (N) were shown. The IHC score of HEY2 in 351 HCC cases was indicated by the box plot (Whiskers, 5-95 percentiles).

To examine the expression of HEY2 expression in HCC, 28 pairs of HCC and the matched nontumourous tissues were collected. Results showed that in 71.4% (20/28) of samples, HEY2 mRNA was markedly overexpressed in HCC tissues, compared to the nontumouous liver tissues (Figure 1A). Consistently, HEY2 protein levels in HCC tissues were noticeably higher in 72.2% (13/18) of cases (Figure 1B). The upregulation of HEY2 in HCC is statistically significant (Figure 1A & 1B). In HCC cell lines, HEY2 mRNA was increased by 5.1-fold on average, compared with the immortalized liver cell line (L-02) (Supplementary Figure S1). Furthermore, result of immunohistochemistry staining in a large cohort of 351 patients with HCC showed a remarkable increase of HEY2 expression in HCC tissues (Figure 1C and Supplementary Figure S2). The percentages of positive immunoactivity in HCC and adjacent tissues were 48.1% and 11.4%, respectively. Overexpression of HEY2 in HCC was observed in 43.6% of samples.

### High HEY2 expression is correlated with unfavorable outcomes in HCC

To determine the clinical significance of HEY2 in HCC, 351 patients were divided into two groups according to the median of IHC score. High HEY2 expression was depicted in 53.3% (187/351) of the HCC cases. Patients with high HEY2 expression were usually companied with multiple (*P*=0.017), poorly differentiated (*P*=0.019), and advanced-stage (*P*=0.006) tumor (Table 1).

**Table 1:** Association of HEY2 expression and clinical features of patients with HCC in training cohort (n=351)

Variable	Hey2 expression			
	All cases	Low expression	High expression	*P* value^a^
Age (years) ^b^				0.811
≤ 47	156	74 (47.4%)	82 (52.6%)	
> 47	195	90 (46.2%)	105 (53.8%)	
Gender				**0.004**
Male	315	139 (44.1%)	176 (55.9%)	
Female	36	25 (69.4%)	11 (30.6%)	
HBsAg				0.712
Negative	53	26 (49.1%)	27 (50.9%)	
Positive	298	138 (46.3%)	160 (53.7%)	
AFP (ng/ml)				0.728
≤ 20	89	43 (48.3%)	46 (51.7%)	
> 20	262	121 (46.2%)	141 (53.8%)	
Cirrhosis				0.303
Yes	301	144 (47.8%)	157 (52.2%)	
No	50	20 (40.0%)	30 (60.0%)	
Tumor size (cm)				0.740
≤ 5	82	37 (45.1%)	45 (54.9%)	
> 5	269	127 (47.2%)	142 (52.8%)	
Tumor multiplicity				**0.017**
Single	221	114 (51.6%)	107 (48.4%)	
Multiple	130	50 (38.5%)	80 (61.5%)	
Differentiation				**0.019**
Well	20	10 (50.0%)	10 (50.0%)	
Moderate	167	90 (53.9%)	77 (46.2%)	
Poor	160	64 (40.0%)	96 (60.0%)	
Undifferentiated	4	0 (0%)	4 (100%)	
Stage				**0.006**
I	119	69 (58.0%)	50 (42.0%)	
II	69	26 (37.7%)	43 (62.3%)	
III	120	46 (38.3%)	74 (61.7%)	
IV	43	23 (53.5%)	20 (46.5%)	
Tumor capsule				0.811
Incomplete	195	90 (46.2%)	105 (53.8%)	
Complete	156	74 (47.4%)	82 (52.6%)	
LNM				0.952
Yes	26	12 (46.2%)	14 (53.8%)	
No	325	152 (46.8%)	173 (53.2%)	
Vascular invasion				0.097
Yes	78	30 (38.5%)	48 (61.5%)	
No	273	134 (49.1%)	139 (50.9%)	

a Chi-square test;

b Median age; AFP, alpha-fetoprotein; HBsAg, hepatitis B surface antigen; LNM, lymph node metastasis.

**Figure 2:**
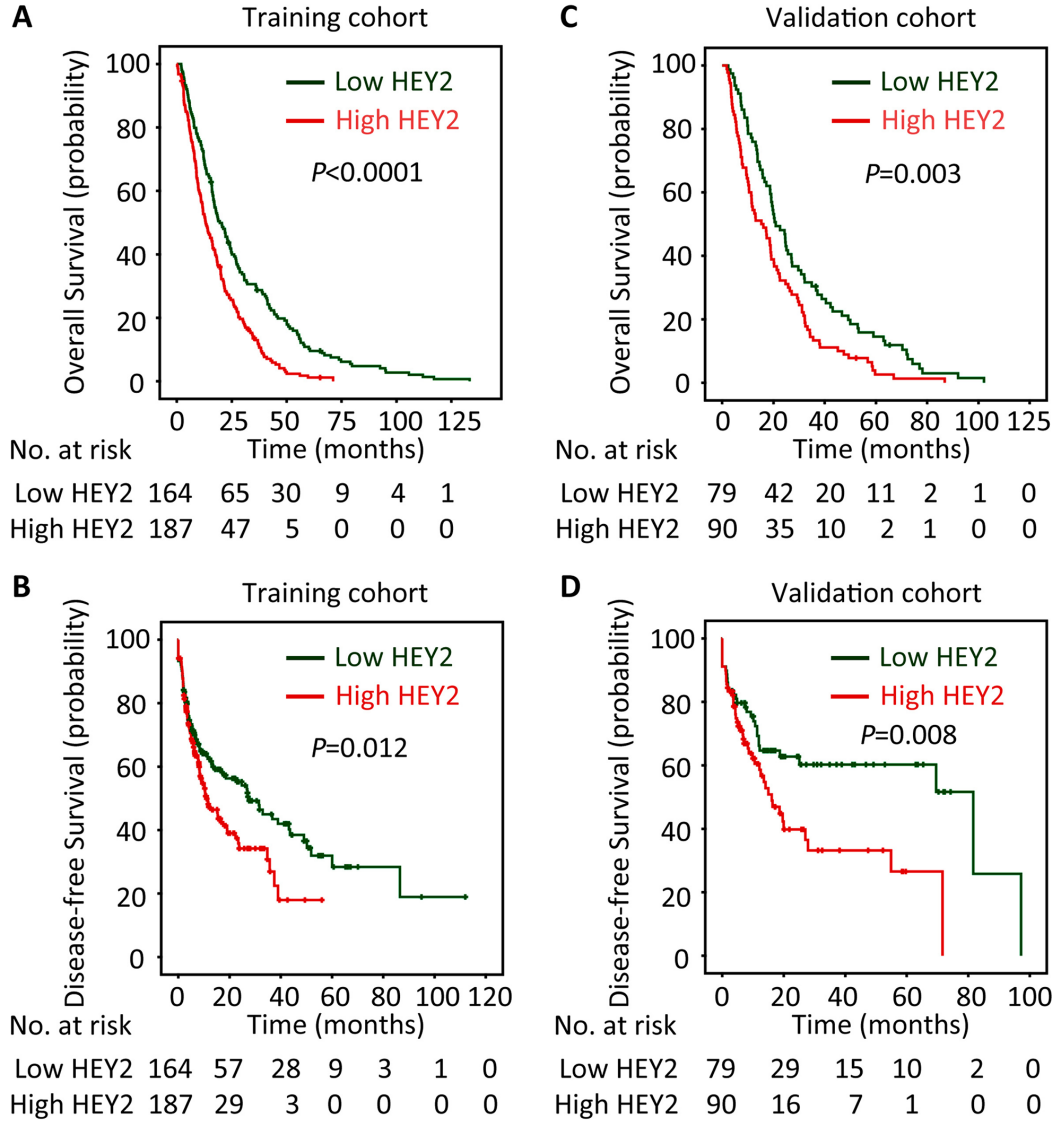
High HEY2 expression is correlated to poor prognosis of patients with HCC According to the median of HEY2 IHC score, patients in training cohort (n=351) and validation cohort (n=169) were separated into two groups named “Low HEY2 expression” and “High HEY2 expression”. The relationship of HEY2 expression and the survival of HCC patients, in terms of overall survival **A**&**C.** and disease-free survival **B**&**D.** was disclosed by Kaplan-Meier analyses (log-rank test).

**Figure 3:**
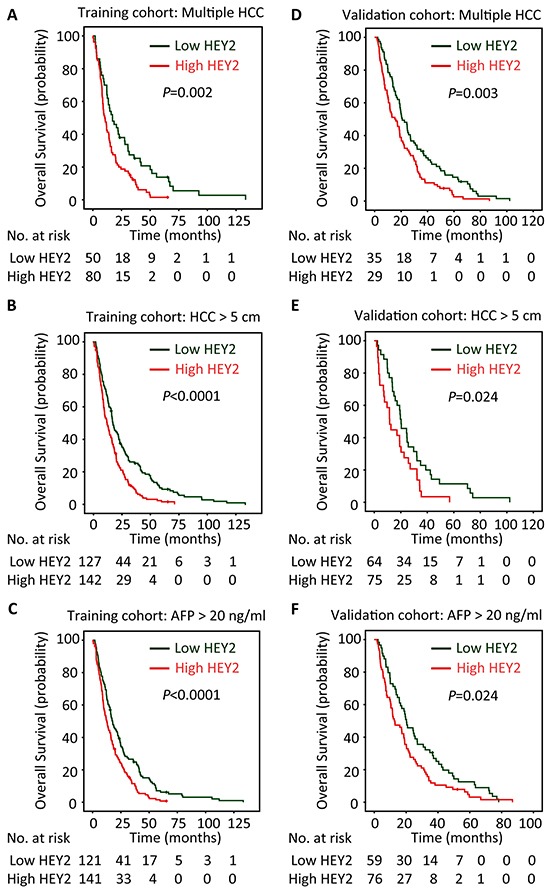
HEY2 expression is associated with overall survival of subgroups of HCC patients in training and validation cohorts The connection of HEY2 expression and overall survival of HCC patients with multiple HCC **A&D.** large HCC **B&E.** and high serum AFP level **C&F.** in both cohorts was determined by stratified survival analysis (log-rank test).

The prognostic value of HEY2 in HCC was also evaluated. In our cohort, 342 deaths were recorded. The 1-year, 3-year and 5-year survival rates are 87.2%, 51.3% and 21.6%, respectively. The median survival was 18.7 and 12.9 months, respectively in high and low HEY2 expression group. Kaplan-Meier analyses revealed that high HEY2 expression was significantly associated with poor overall survival (*P*<0.0001) (Figure 2A) and disease-free survival (*P*=0.012) (Figure 2B). This was further confirmed in a validation cohort of 169 HCC patients. The 1-year, 3-year and 5-year survival rates in validation cohort are 90.2%, 56.8% and 26.5%, respectively. Patients expressing less HEY2 survived longer (*P*=0.003) (Figure 2C) and experienced a longer period without tumor progression (*P*=0.008) (Figure 2D). Stratified survival analyses validated the prognostic value of HEY2. In the subgroups (tumor size, AFP level, tumor differentiation, TNM stage), HEY2 expression was reversely associated with the survival of post-resection HCC patients (Figure 3 and Supplementary Figure S3).

Univariate analyses indicated HEY2, as well as tumor size, AFP, tumor capsule, TNM stage, vascular invasion, lymph node metastasis and tumor differentiation, served as a prognostic factor in HCC (Table 2). Multivariate analyses revealed that HEY2 was of independent implication in prediction of overall survival (hazard ratio (HR) = 1.645, 95% confident interval (CI): 1.309-2.067, *P* < 0.001) (Table 2).

**Table 2:** Univariate and multivariate analysis of HEY2 expression and outcomes of HCC patients in training cohort (n=351)

Variables	Univariate analysis		Multivariate analysis	
	HR (95% CI)	*P* value	HR (95% CI)	*P* value
Age (≤ 47 vs. > 47 years)	0.796 (0.642-0.988)	0.138		
Gender (female vs. male)	0.846 (0.593-1.207)	0.357		
HBsAg (positive vs. negative)	1.175 (0.870-1.587)	0.294		
Tumor size (≤ 5 vs. > 5 cm)	1.374 (1.069-1.765)	**0.013**	1.316 (1.007-1.719)	**0.045**
Tumor multiplicity (single vs. multiple)	1.215 (0.974-1.517)	0.085		
Tumor capsule	0.648 (0.521-0.807)	**0.000**	0.706 (0.558-0.895)	**0.004**
Liver cirrhosis (yes vs. no)	0.698 (0.514-0.950)	**0.022**	0.765 (0.558-1.048)	0.095
AFP (≤ 20 vs. > 20 ng/mL)	1.388 (1.086-1.773)	**0.009**	1.159 (0.899-1.495)	0.254
LNM (Yes vs. no)	1.816 (1.213-2.717)	**0.004**	1.588 (1.042-2.420)	**0.032**
Tumor differentiation	1.431 (1.202-1.704)	**0.000**	1.266 (1.055-1.521)	**0.011**
TNM (I vs. II vs. III vs. IV)	1.237 (1.123-1.362)	**0.000**	1.081 (0.967-1.209)	0.172
Vascular invasion (Yes vs. No)	1.808 (1.393-2.346)	**0.000**	1.215 (0.911-1.621)	0.185
HEY2 expression (low vs. high)	1.753 (1.401-2.193)	**0.000**	1.645 (1.309-2.067)	**0.000**

HR, hazard ratio; CI, confident interval; AFP, alpha-fetoprotein; HBsAg, hepatitis B surface antigen; LNM, lymph node metastasis.

### HEY2 promotes cell proliferation and migration in HCC

**Figure 4:**
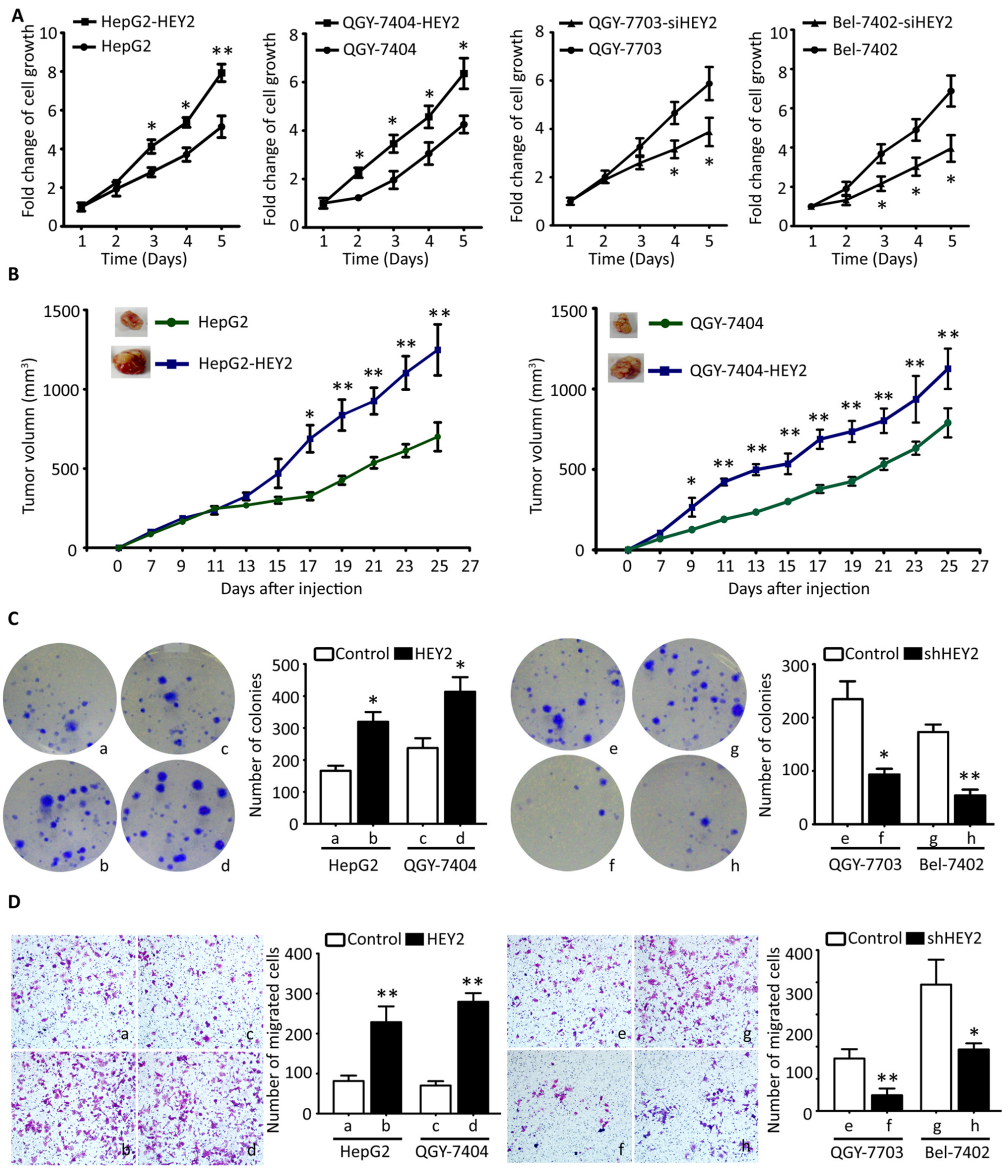
HEY2 promotes cell proliferation and migration in HCC cells **A.** Cells transfected by HEY2 overexpression vector (HepG2 and QGY-7404) or HEY2 siRNA (QGY-7703 and Bel-7402) were cultured for 5 days. The cell viabilities were determined by MTT assays. The fold changes of cell growth were calculated and indicated by curves. **B.** Colony formation was used for determination of HEY2-mediated cell proliferation. Stable cell lines with HEY2 overexpression or knockdown were incubated for 14 days. The number of colonies were counted and shown by histogram. **C.** Cells were injected into the right flank of mice. The tumor size was measured every other day. The tumor growth was indicated by curves. **D.** Transwell assays were performed to evaluate the effect of HEY2 on cell migration *in vitro*. Stable cells were cultured for 48 hours. The number of migrated cells were counted and shown. All data are mean ± SEM. *P <0.05, **P<0.01.

To investigate the effect of HEY2 on cell growth in HCC, HEY2 was overexpressed or knocked down. MTT and BrdU assays showed that HEY2 overexpression in HepG2 and QGY-7404 cells increased, whereas HEY2 knockdown in QGY-7703 and QGY-7402 cells decreased the cell viabilities and proliferation (Figure 4A and Supplementary Figure S4). Colony formation assays revealed that the number of foci formed by HEY2-expressed cells was increased by twice, compared to the control. Clone production was inhibited in HEY2-depleted cells (Figure 4B). *In vivo* data showed that overexpression of HEY2 significantly accelerated the growth of xenografts (Figure 4C). Transwell assays indicated that the cell migration was enhanced by HEY2 overexpression, but attenuated by HEY2 knockdown in HCC cells (Figure 4D).

### *HEY2* is a direct target of miR-137

**Figure 5:**
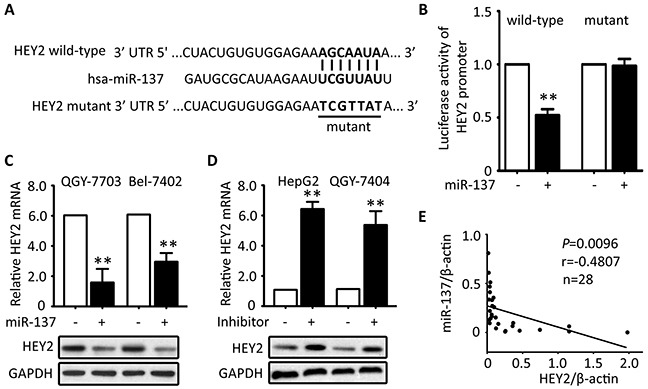
HEY2 expression is inhibited by miR-137 in HCC cells **A.** Sequence of 3′-UTR of HEY2 promoter and the matched 7-bp sequence were shown. The schematic constructions of wild-type and mutant pGL3-HEY2 vectors were indicated. **B.** The relative luciferase activities modulated by miR-137 were analyzed in Bel-7402 cells. **C.** miR-137 was overexpressed in QGY-7703 and Bel-7402 cells for 48 hours. The mRNA and protein levels of HEY2 were examined by qRT-PCR and Western blot. **D.** The inhibitor of miR-137 was introduced into HepG2 and QGY-7404 cells for 48 hours. The mRNA and protein levels of HEY2 were determined. **E.** The correlation of miR-137 and HEY2 mRNA in 18 fresh HCC tissues were calculated and indicated. All data are mean ± SEM. **P <0.01.

To disclose the mechanism of HEY2 overexpression in HCC, prediction analyses were conducted in searching programs (Targetscan, miRtarget and PITA). HEY2 was predicted as a direct target of miR-137. Vectors encoded wild-type or mutant 3′UTR of *HEY2* were established by fusing with the firefly luciferase gene (Figure 5A). Results showed that overexpression of miR-137 significantly reduced the relative luciferase activity of wild-type *HEY2* promoter but not of the mutant (Figure 5B). Re-introduce of miR-137 into QGY-7703 and Bel-7402 cells led to markedly decreased levels of HEY2 mRNA and protein (Figure 4C). In contrast, miR-137 inhibitor induced the mRNA and protein expression of HEY2 in HepG2 and QGY-7404 cells (Figure 5D). In clinical samples, miR-137 expression was reversely associated with the *HEY2* mRNA expression in 18 HCC fresh tissues (Figure 5E).

## DISCUSSION

In the last decades, efforts have been made to identify biomarkers suitable and useful for early diagnosis and efficient treatment. Plenty of potential factors have been suggested as promising targets for clinical management. In this study, we found that in two independent cohorts, HEY2 expression was markedly increased. High HEY2 expression was significantly correlated with advanced stage and poor tumor differentiation. Furthermore, HEY2 served as an independent factor for overall outcome of patients in HCC.

In human cancers, HEY2 overexpression exerts pro-oncogenic activities. Qu et al. showed that decrease of HEY2 mediated by Notch2 depletion contributed to the attenuation of cell proliferation and migration in salivary adenoid cystic carcinoma [14]. HEY2 increase induced by Notch activation contributed to the acquisition of chemoresistance in gastric cancer [15]. Our data demonstrated that overexpression of HEY2 increased cell viabilities, colony formation and cell migration, whereas knockdown of HEY2 resulted in the opposite phenotypes. This indicates that HEY2 functions as an oncogene in HCC by promoting cell proliferation and migration. Our data was supported by the other studies. Gao et al. reported that HEY2 upregulated by hepatocyte growth factor (HGF) promoted femoral artery endothelial cell growth [16]. Wu et al. reported that HEY2 was capable of modulating the expression of MMP10 [17]. Wöltje and colleagues reported that HEY2 expression was induced by serum stimuli via the activation of BMP-Smad signaling which might promote HCC cell growth [18, 19]. The interactions of HEY2 with Stat3 [20] and Id4 [21] that play important roles in tumorigenesis, also confirm the oncogenic role of HEY2 in human cancers.

HEY2 plays an important role in cell differentiation. HEY2 ortholog gridlock was induced by Sox7 and Sox18 during zebrafish arteriovenous differentiation [22]. Rowlinson et al. showed HEY2 was required for the formation of hematopoietic stem cell via acting as the upstream of Notch [23]. In the present study, HEY2 expression was associated with tumor differentiation. High expression of HEY2 was presented in all of the undifferentiated HCC, indicating that HEY2 may block the cell differentiation. This may be supported by the study that showed HEY2 was capable of maintaining the stem cell characteristics of neural precursors [24].

HEY2 was revealed as a direct target of miR-137 that is frequently downregulated in human cancers including HCC. In HCC samples, HEY2 expression was inversely correlated to miR-137 expression. To date, another four genes (FOXO1 [25], AKT2 [26], CDC42 [27] and EDIL3 [28]) have been shown to be inhibited by miR-137. Interestingly, these five genes (HEY2, FOXO1, AKT2, CDC42 and EDIL3) were implicated in PI3K/Akt pathway. Whether HEY2 could interact with the other proteins required further investigations. Furthermore, miR-137 has been demonstrated to inhibit neuronal maturation and dendritic morphogenesis [29], and to associate with unfavorable prognosis [26]. It should be of clinical significance to assess the prognostic implication of the combination of miR-137 and HEY2 expressions. Our study therefore suggests HEY2 as a promising biomarker for unfavorable outcomes and a novel therapeutic target for the clinical management of HCC.

## MATERIALS AND METHODS

### Patients and tissue specimens

A training cohort consisting of 351 paraffin-embedded tissues and complete clinical and pathological data were collected from HCC patients who received surgical resection at The Third Affiliated hospital of Sun Yat-sen University, Guangzhou, China, between March 2008 and April 2010. A validation cohort containing 169 patients with HCC and the corresponding clinical data were collected at The Affiliated Hexian Memorial Hospital of Southern Medical University, between Jan 2005 and Dec 2011. All the tissues in both cohorts were used for the construction of tissue microarray (TMA), according to our previous study [30]. Another 18 pairs of fresh HCC tissues and the corresponding adjacent nontumorous tissues were obtained for qRT-PCR and Western blot. None of the patients had received adjuvant therapies before surgery. This study was approved by the Institute Research Medical Ethics Committee of The Third Affiliated hospital of Sun Yat-sen University. No informed consent (written or verbal) was obtained for use of retrospective tissue samples from the patients within this study, since this was not deemed necessary by the Ethics Committee.

### Quantitative reverse-transcription PCR (qRT-PCR)

Total mRNA extracted from the fresh samples was reversed to cDNA by M-MLV Reverse Transcriptase (Promega Inc., USA). Levels of HEY2 and β-actin were measured by SYBR green-based real-time PCR using the Stratagene Mx3000P Real-Time PCR system. Primers were designed as follows: HEY2, forward: 5′- TGGGGAGCGAGAACAATTAC-3′ and reverse: 5′-TTTTCAAAAGCTGTTGGCACT-3′; β-actin, forward: 5′-TGGCACCCAGCACAATGAA-3′ and reverse: 5′-CTAAGTCATAGTCCGCCTAGAAGCA-3′. The relative expression of HEY2 was normalized to the β-actin, using the comparative threshold cycle (2^−ΔCt^) method.

### Western blot

Total proteins extracted from HCC fresh tissue were fractionated by SDS-PAGE, transferred to PVDF membrane, and then incubated with a primary specific antibody for HEY2 (1:1500, Simga: HPA030205) in 5% of non-fat milk, followed by a horse radish peroxidase (HRP)-conjugated anti-rabbit second antibody. ECL detection reagent (Amersham Life Science, Piscataway, NJ, USA) was used to show the results.

### Immunohistochemistry (IHC)

TMA sections with a thickness of 4 μm were dewaxed in xylene and graded alcohols, hydrated, and washed in phosphatebuffered saline (PBS). After pretreatment in a microwave oven, endogenous peroxidase was inhibited by 3% hydrogen peroxide in methanol for 20 min, followed by avidin-biotin blocking using a biotin-blocking kit (DAKO, Germany). Slides were then incubated with HEY2 antibody (1:400, Simga: HPA030205), overnight in a moist chamber at 4°C, washed in PBS, and incubated with biotinylated goat anti-rabbit antibody. Slides were developed with the Dako Liquid 3,′3-diaminobenzidine tetrahydrochloride (DAB) + Substrate Chromogen System and counterstained with hematoxylin.

### IHC evaluation

HEY2 protein level was determined by semi-quantitative IHC detection, using the H-score method. The percentage of positively-stained cells was scored as “0” (0%), “1” (1%-25%), “2” (26%-50%), “3” (51%-75%), “4” (76%-100%). Intensity was scored as “0” (negative staining), “1” (weak staining), “2” (moderate staining), and “3” (strong staining). The percentage score was multiplied by the staining intensity score. For each case, 1000 cells were randomly selected and scored. The scores were independently decided by 2 pathologists (SG Su and D He). The median of HEY2 IHC score, which was 2.5, was chosen as the cutoff value to identify high and low HEY2 expression in HCC tissues.

### Luciferase reporter assay

For the binding of miR-137 to HEY2 3′ UTR, Bel-7402 cells were co-transfected with miR-137 mimic or negative control and wild-type or mutant vectors for 36 hours. The relative luciferase activity was analyzed with the Dual-Luciferase Reporter Assay System (Promega, CA, USA).

### MTT

Cells with HEY2 overexpression or knockdown were cultured in 96-well plates for 5 days. At the indicated time, 100 μL of MTT was added for 2.5 hours. 100 μL of DMSO was then added to solve the crystal. Finally, density was measured at 490 nm with background subtraction at 670 nm by Model 550 Microplate Reader (Bio-Rad, Hercules, CA, USA). The fold change of cell growth was calculated based on the absorbance of 490 nm.

### Colony formation

Cells were plated into 6-well plates at a density of 500 cells per well. After 10 days of incubation in complete culture medium, the cells colonies were fixed and stained with crystal violet solution (0.1% crystal violet) for 10 min, washed with PBS, and counted.

### Transwell assay

Two thousands of cells were placed in the upper compartment of a Transwell chamber for migration assay. DMEM medium with 15% fetal bovine serum was filled into the lower chamber. After 48 hours, the cells on the upper surface of the membrane were removed, and the cells on the lower surface were fixed with methanol and stained with 0.1% crystal violet and counted. Six visual fields were randomly chosen.

### Animal model

1×10^7^ cells were injected into the right flank of 4-week aged null mice. Tumors were measured every other day to estimate the volume from day 7 to day 27 after injection. Tumor sizes were evaluated using the formula: Volume (mm3) = [width^2^ (mm^2^) × length (mm)]/2.

### Statistical analysis

Statistical analyses were performed using the SPSS 19.0 software (SPSS, Chicago, IL, USA). Wilcoxon matched paired test was used to determine the significance of HEY2 expression in fresh HCC and normal liver tissues. χ^2^ test was performed to analyze the correlation between HEY2 expression and clinicopathological parameters. Kaplan-Meier analysis (log-rank test) was utilized for survival analysis. Cox proportional hazards regression model was used to identify the independent prognostic factors. *P*<0.05 (two-tailed) was considered statistically significant.

## SUPPLEMENTARY FIGURES


