# Supplementary material for: HEY2, a target of miR-137, indicates poor outcomes and promotes cell proliferation and migration in hepatocellular carcinoma
Source: Oncotarget. 2016 May 13;7(25):38052–63. doi: 10.18632/oncotarget.9343 (PMC5122371; doi:10.18632/oncotarget.9343)
Supplement: Supplementary file 1 [file oncotarget-07-38052-s001.pdf]

SUPPLEMENTARY FIGURES

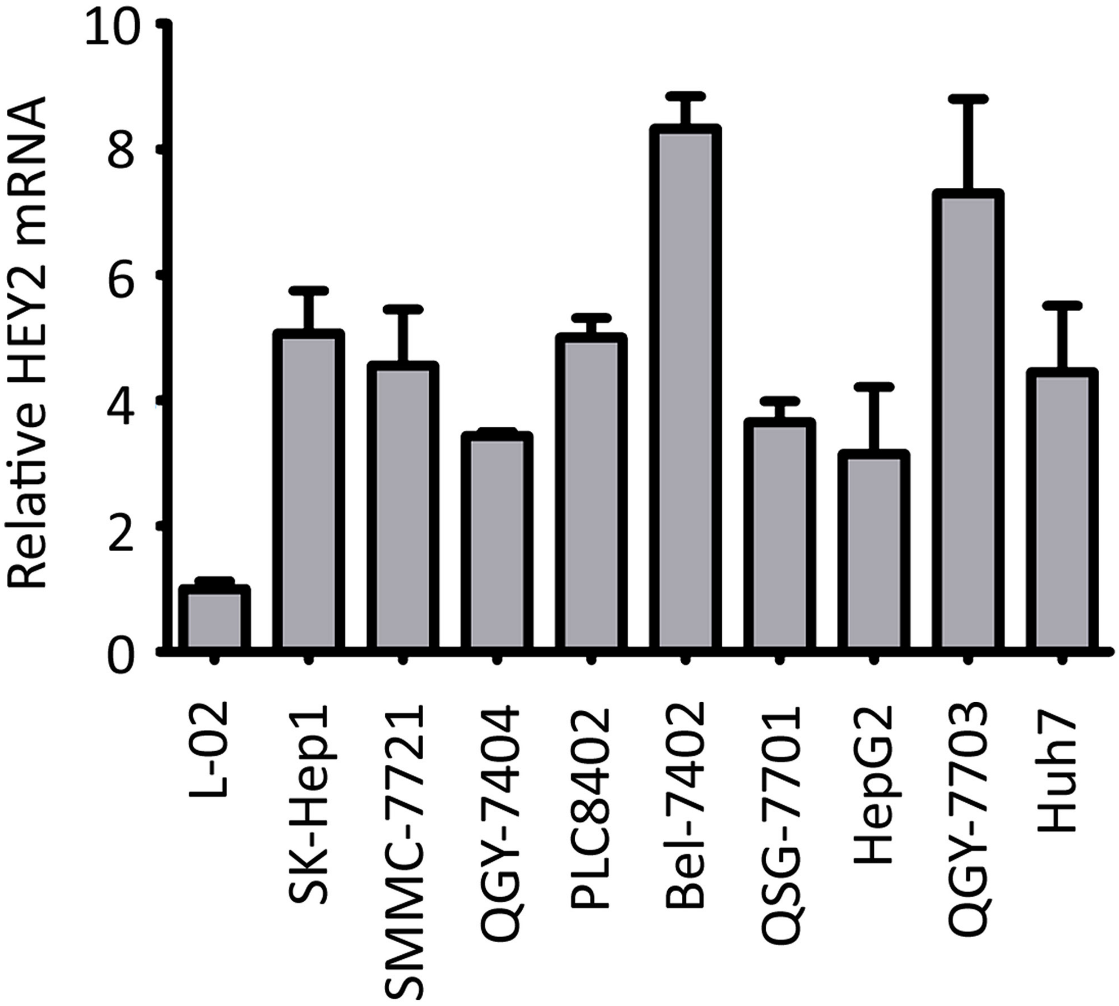

Supplementary Figure S1: HEY2 mRNA expression in HCC cell lines. The expressions of HEY2 mRNA in HCC cell lines (HepG2, QGY7404, Bel-7402, QGY-7703, Huh7, QSG-7701, PLC8402, SMMC-7721 and SK-Hep1) and immortalized liver cell line (L-02) were determined by qRT-PCR.

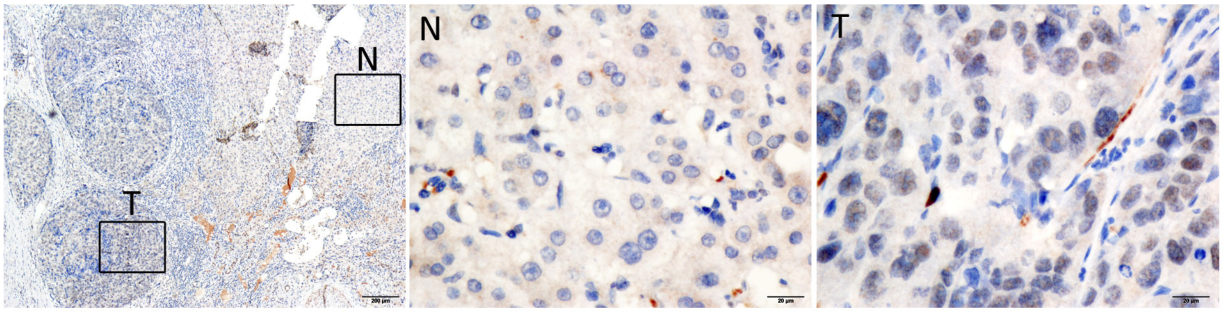

Supplementary Figure S2: HEY2 expression in tumor and nontumor tissues on the same slide. IHC staining was performed on the slide that consisting of both HCC and nontumorous tissues.

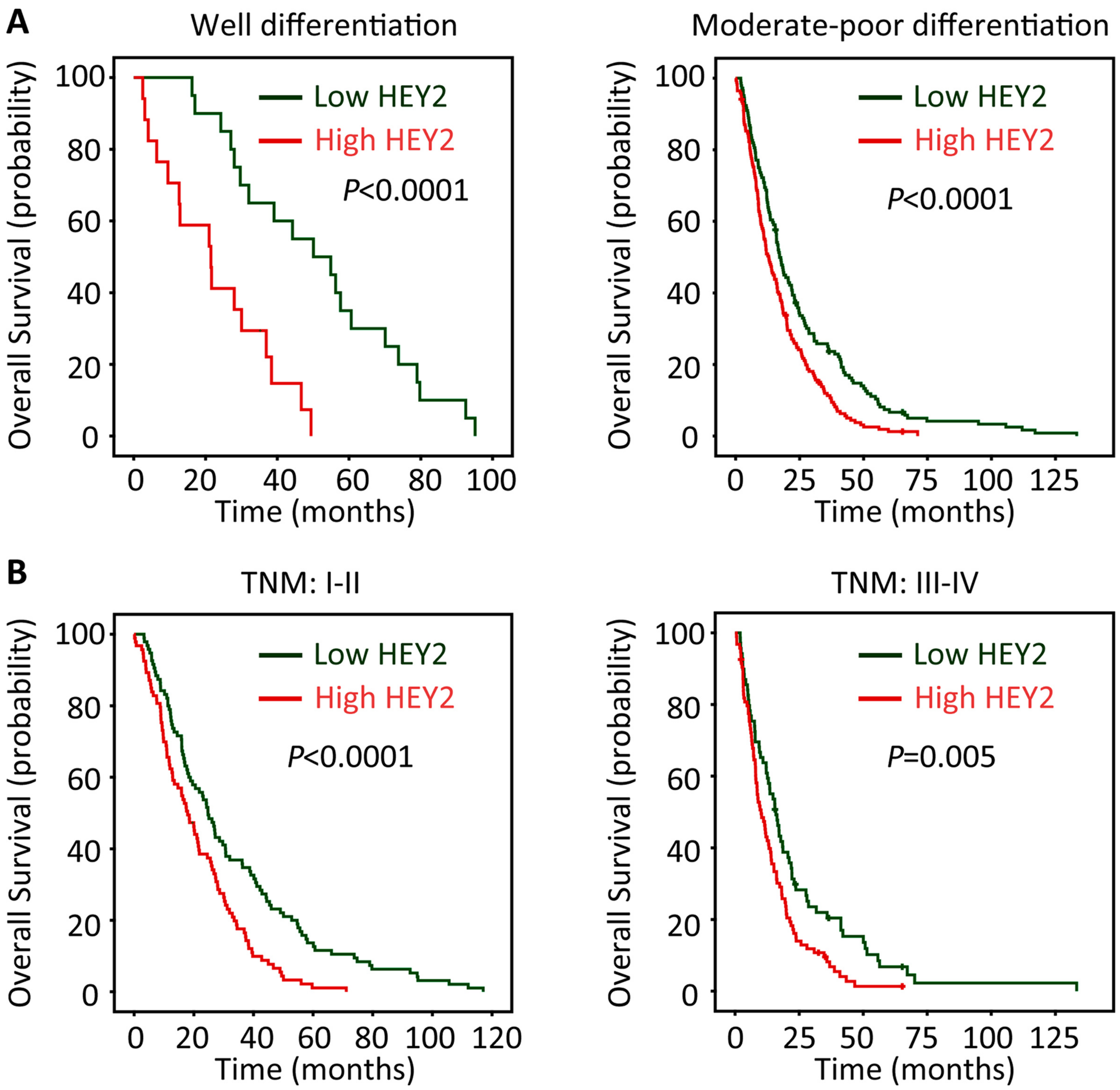

Supplementary Figure S3: The prognostic value of HEY2 in patients with different tumor differentiation and TNM stage in training cohort. The connection of HEY2 expression and overall survival of HCC patients with well or moderate-poor tumor differentiation **A.** and I-II or III-IV TNM stage **B.** was determined by stratified survival analysis (log-rank test).

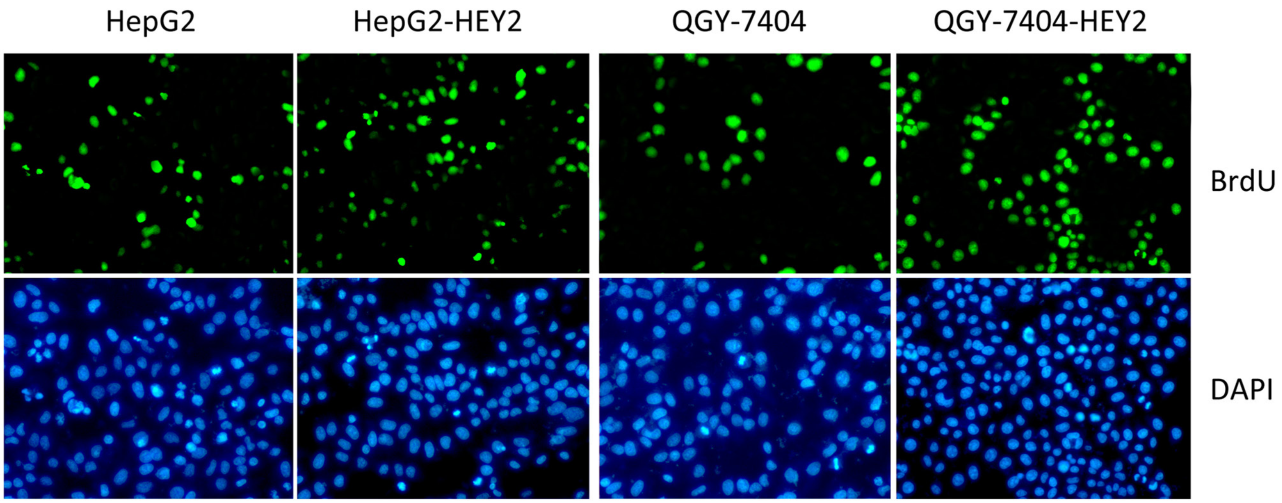

Supplementary Figure S4: Effect of HEY2 overexpression on cell proliferation. Cells were transfected with HEY2 overexpression or empty vectors for 24 h. BrdU assays were performed to evaluate the effect of HEY2 overexpression on cell proliferation.
